# Application of 128-slice spiral CT combination scanning in the diagnosis of embolisms in pulmonary arteries and lower extremity veins

**DOI:** 10.3892/etm.2013.1424

**Published:** 2013-11-25

**Authors:** LINYOU WANG, WUGEN KANG, MAOHENG ZU, QINGQIAO ZHANG, JIANMIN SHEN, LIANG WU, DONGGUO WANG

**Affiliations:** Department of Radiology and Intervention, Municipal Hospital of Taizhou, Taizhou, Zhejiang 318000, P.R. China

**Keywords:** pulmonary embolism, deep vein thrombosis, diagnosis, spiral computed tomography, digital subtraction angiography

## Abstract

The aim of this study was to evaluate the significance of using multi-row spiral computed tomography (CT) to scan for pulmonary artery thrombosis and lower limb deep vein thrombosis (LVT) in patients with suspected LVT. A total of 110 patients underwent a contrast-enhanced spiral CT inspection of the pulmonary artery and lower extremity veins. Three-dimensional digital image processing, including multi-planar reconstruction (MPR), maximum intensity projection (MIP) and volume rendering (VR), was also conducted; two groups of experienced radiologists analyzed the CT images to evaluate the postprocessing techniques of these CT images. Seventy-five patients were diagnosed with LVT with or without pulmonary embolism (PE); out of these 75, 34 patients were diagnosed with PE and LVT together and 41 patients were diagnosed with LVT alone. A further 31 patients were diagnosed with iliac vein compression syndrome (IVCS), and no embolisms were detected in the remaining four patients. With regard to PE, MPR and MIP demonstrated an accuracy of 100%, while MPR also showed images of LVT with an accuracy of 100%. The follow-up results at 12 months were consistent with the CT scan results. The clinical use of 128-slice spiral CT combination scanning in the detection of PE and LVT has significant potential to improve upon the present methods of diagnosis.

## Introduction

Venous thromboembolic diseases comprise pulmonary embolism (PE) and deep venous thrombosis (DVT) (1–3); untreated DVT may lead to a potentially fatal PE (4). DVT and PE have increasingly been considered as a single disease, known as venous thromboembolism (VTE) (5), and an asymptomatic PE is present in approximately half the patients presenting with symptomatic proximal DVT (6). Moreover, DVT and PE share a number of risk factors, including age, immobilization, major surgery or trauma, active cancer, pregnancy, oral contraceptive use and hormone replacement therapy. Iliac vein compression syndrome (IVCS) remained relatively unknown until 1957, when May and Thurner (7) characterized three types of intraluminal bands, or ‘spurs’ within the compressed iliac vein that were hypothesized to be probable risk factors for the development of left-sided iliofemoral DVT (7).

With the advent of catheter-directed thrombolysis, iliac vein compression has been observed to be frequently associated with DVT following iliofemoral vein thrombolysis (8). The management of iliofemoral DVT remains a challenge due to the fact that the symptoms and signs of DVT are unspecific. It has been shown that <25% of patients with clinically suspected DVT actually have the disease (9,10), which emphasizes the importance of accurate diagnostic strategies. The correct diagnosis and prompt treatment are therefore crucial. Several clinical prediction rules have been developed to simplify and improve the diagnostic procedures for patients with suspected DVT in a number of populations (11–15).

Diagnostic strategies based on combining pretest probability with D-dimer measurements have been shown to be safe and cost-effective (16), leading to a significant reduction in the number of ultrasound examinations (12,17,18). As a result of the ability to acquire processed data sets with spiral computed tomography (CT), different techniques are available for accurate diagnosis. A series of robust and reproducible measurements for DVT is likely to be beneficial for the establishment of spiral CT as a clinical tool.

The aim of the present study was to assess the optimal digital image processing and combination techniques for the diagnosis of DVT, PE and IVCS, and to evaluate their accuracy.

## Patients and methods

### Patients

Although this examination was performed for accepted clinical indications and was considered suitable for patient care, approval was obtained from the institutional review board of the Municipal Hospital of Taizhou (Taizhou, China). Informed consent was obtained from each patient once the nature of the procedure had been explained fully.

A cohort of 110 consecutive patients (47 males and 63 females; mean age, 55±9 years; range, 27–84 years) was recruited from January 2010 to April 2012. All patients were suspected to have lower limb deep vein thrombosis (LVT) following B-mode ultrasonography. The patient population was composed of inpatients and outpatients whose physicians had ordered combined pulmonary CT and lower limb angiography, as well as indirect CT venography (CTV), for the diagnosis of VTE. For patients with no IVCS, an inferior vena cava filter was implanted prior to interventional treatment in order to reduce the further risk of DVT or PE.

### CT acquisition protocol

All coronary CT angiographic examinations were performed on a 128-slice spiral CT scanner (GE LightSpeed 7.0 CT Scanner System; GE Medical Systems, Waukesha, WI, USA). The patients were scanned in the lateral position, with their feet placed into the CT scanner first. On the basis of the patients’ weights, 120–150 ml (2 ml/kg) nonionic contrast medium (Optiray 350; Tyco Healthcare, Montreal, QC, Canada) was injected into the antecubital vein at a mean flow rate of 4 ml/sec using a high-pressure syringe. This was followed by a chaser bolus of 30 ml saline at the same flow rate using a dual-head injector (Stellant^®^ D Dual Syringe CT Injection System; Medrad, Warrendale, PA, USA). To optimize the starting time for acquisition, a contrast agent auto-tracking technique was used (19). A prescan was performed at the level of the aortic root, and a circular region of interest measuring 10 mm in diameter was placed on the ascending aorta. As soon as the signal density in the region of interest was obtained, image acquisition was initiated.

A spiral pulmonary CT angiography (PCTA) check was performed, prior to a CTV being conducted 2 min later, combined with the time-density curves (20,21). All image data were processed by Wizard workstation (GE advantage windows 4.0; GE Healthcare, Wood Dale, IL, USA), including multi-planar reconstruction (MPR), maximum intensity projection (MIP) imaging and volume rendering (VR).

These digital subtraction angiography (DSA) techniques were used to diagnose thrombosis of the pulmonary blood vessels, LVT and IVCS. The pulmonary subsegments and branches were further observed by adjusting the window width and level from 40 to 80 HU (22,23). The examinations were preselected for adequate contrast enhancement of the pulmonary arteries, which was judged subjectively.

### CT image postprocessing and results analysis

Two groups of radiologists (experienced attending physicians, practicing for >10 years, three in each group) read the imaging results; the PCTA and CTV image results were read by the radiologists in group 1, and then the postprocessing techniques of MPR, MIP and VR were conducted for each image. The processed images were read by the second group of radiologists. According to the interpretation of the results, the patients were diagnosed with thrombosis of the pulmonary blood vessels, LVT and IVCS by the reviewers. The detection results of group 1 were considered as the standard to assess the accuracy of the image processing in group 2. In addition, a 12-month follow-up with PCTA and CTV was conducted to evaluate the credibility of the diagnoses.

## Results

### Enhancement CT diagnosis results

The enhancement CT value of the normal pulmonary artery in our hospital (Municipal Hospital of Taizhou) was 270±22 HU and the main pulmonary artery and its branches were uniformly distributed on the image. The CT value of the pulmonary artery embolism was 65±7 HU and the image showed typical filling defects within the vascular cavity, which were clearly revealed by PCTA. The enhancement CT value of the normal lower extremity vein was 115±11 HU, while that of the LVT was 70±7 HU. The image of the LVT showed a filling defect.

**Figure 1:**
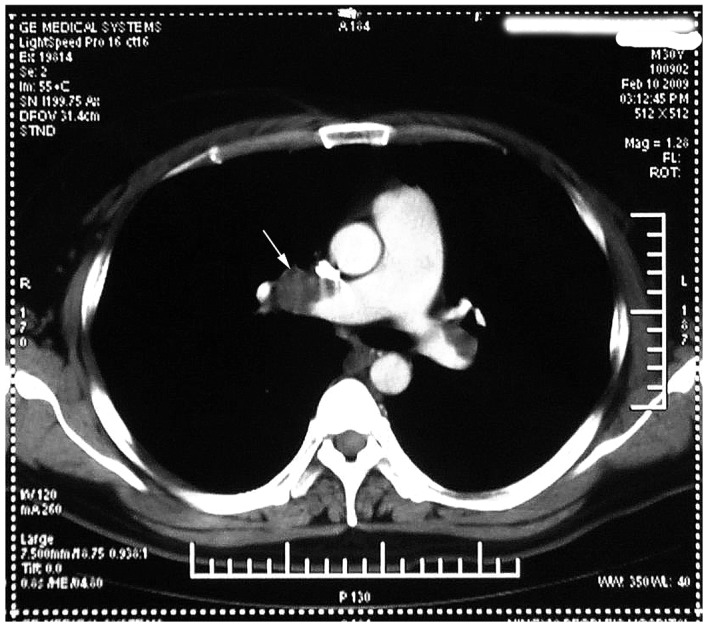
Original pulmonary artery cross-sectional image, showing the typical filling defect (arrow) of pulmonary thrombosis.

Following the diagnostic procedure, 75 out of the 110 patients were diagnosed with LVT; IVCS was observed in 31 patients; and four patients were negative for embolisms. Out of the 75 patients diagnosed with LVT, 34 patients also presented with PE. In the patients with IVCS, the thrombosis extended to the iliac vein, inferior vena cava and renal vein in 10 of the 31 patients. Fig. 1 shows the filling defect within the pulmonary vascular cavity of an unprocessed CT image.

### Credibility results of the postprocessed images

**Figure 2:**
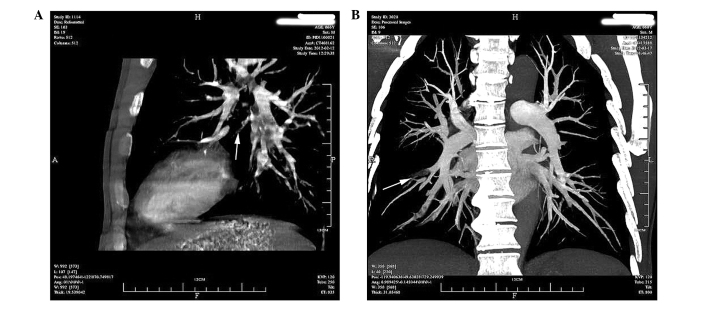
Multi-planar reconstruction (MPR) image of a right pulmonary artery. (A) Central pulmonary artery filling defect; (B) lower pulmonary branch filling defect.

**Figure 3:**
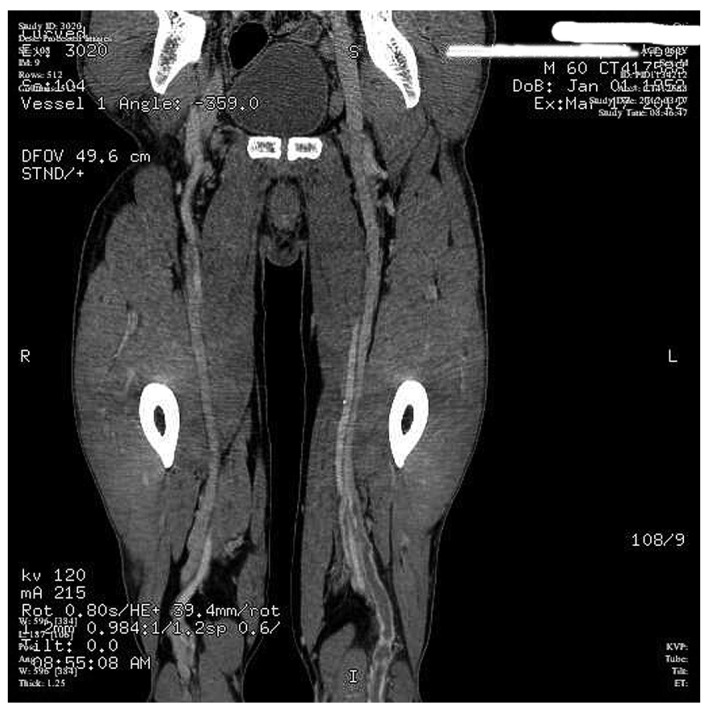
Volume rendering (VR) image of a left lower limb deep vein, which shows developed venous limitations of thrombosis.

When the credibilities of the three modes of image postprocessing were compared, as shown in Table I, compared with the VR processing technology, MPR and MIP were more effective at showing thrombosis in the pulmonary artery, and clearly revealed the presence and range of the thrombosis. Fig. 2 shows two MPR images of the right pulmonary artery, in which the central artery (Fig. 2A) and lower pulmonary branch (Fig. 2B) show filling defects. Due to the concentration of the contrast medium, MIR and VR (Fig. 3) showed the thrombosis image of the lower limb deep vein clearly when combined with the original CT image.

Compared with the display rate of the original CT image (100%), the display rates of MPR, MIP and VR were 100% (34/34), 100% (34/34) and 65% (22/34) for PE with LVT; 100% (41/41), 61% (25/41) and 49% (20/41) for LVT alone; and 100% (31/31), 100% (31/31) and 100% (31/31) for IVCS, respectively. MPR was a more effective DSA technique than MIP and VR in the present evaluation, and the difference was statistically significant (P=0.001).

All 75 patients finished the outpatient follow-ups 12 months later, and the CT follow-up results confirmed the diagnosed results.

## Discussion

Traditional lower extremity studies that assess and review the entire lower extremity vasculature are performed by an ultrasound technologist. However, ultrasound examinations are not always available and have been shown to delay the time to diagnosis and potential treatment of a DVT by ~2 h (24,25). The ‘one-stage’ examination for the pulmonary artery and lower limb deep veins simultaneously, using high-speed spiral CT imaging technology, significantly reduces the total dose of contrast agent used. The procedure is relatively simple (pulmonary scanning time, 6 sec; time of moving patients, 5 sec; lower limb deep vein scanning time, 15–20 sec), and offers a convenient option for ambulatory patients.

The conventional time-delay for a PCTA inspection is 15–17 sec (26). The contrast agent auto-tracking technology is able to correctly evaluate the delay-time. However, the time-delay range for a lower extremity CTV is relatively longer and measures 120–150 sec, depending on the condition of the patients, with a delay of 150 sec in cases of cardiac insufficiency or varicose veins of the lower extremity and a delay of 120 sec in cases without dysfunction or varicose veins. In the present study, the time-density curves combined with MPR images clearly showed the LVT in the 75 diagnosed patients.

The production of near-isotropic data sets with 128-slice spiral CT has enabled the introduction and/or refinement of numerous image processing techniques, avoiding the inherent distortion associated with non-isotropic data. CTV of the iliac vein is capable of effectively assessing the nature of thrombosis, particularly for the diagnosis of IVCS. The correct diagnosis contributes to the correct treatment, in addition to reducing unnecessary economic burden on the patients.

The advantages of 128-layer spiral pulse CT scanning are that it is noninvasive, scans at a high speed and generates images simultaneously. The results of the present study demonstrated that the diagnosis of LVT using 128-slice spiral CT combination scanning was accurate when compared with the original CT image. In the diagnosis of PE, the DSA techniques of MPR and MIP showed the image clearly, while MPR also clearly displayed the image of LVT. Combined with the original images, MIP and VR were able to diagnose LVT efficiently, while all of the three DSA techniques showed the images of IVCS clearly. This novel scanning technique has significant potential to improve upon the present diagnosis and management of patients with LVT.

## Figures and Tables

**Table I:** Credibility of diagnostic results from postprocessed images compared with the results of direct CT in 110 patients.

		Postprocessing positive rate, n (%)			
			
PCTA/CTV	Condition	MPR	MIP	VR	Positive by direct CT, n^a^
PCTA	PE+LVT	34 (100)	34 (100)	22 (65)	34
CTV	LVT	41 (100)	25 (61)	20 (49)	41
CTV	IVCS	31 (100)	31 (100)	31 (100)	31

a Four patients were negative for embolism by direct CT.

CT, computed tomography; PCTA, pulmonary CT angiography; CTV, CT venography; PE, pulmonary embolism; LVT, lower venous thrombosis; IVCS, iliac vein compression syndrome; MPR, multi-planar reconstruction; MIP, maximum intensity projection; VR, volume rendering.

## References

[1] Perrier A, Roy PM, Sanchez O (2005). Multidetector-row computed tomography in suspected pulmonary embolism. N Engl J Med.

[2] Elias A, Cazanave A, Elias M (2005). Diagnostic management of pulmonary embolism using clinical assessment, plasma D-dimer assay, complete lower limb venous ultrasound and helical computed tomography of pulmonary arteries. A multicentre clinical outcome study. Thromb Haemost.

[3] Wildberger JE, Mahnken AH, Das M, Küttner A, Lell M, Günther RW (2005). CT imaging in acute pulmonary embolism: diagnostic strategies. Eur Radiol.

[4] Engelberger RP, Aujesky D, Calanca L, Staeger P, Hugli O, Mazzolai L (2011). Comparison of the diagnostic performance of the original and modified Wells score in inpatients and outpatients with suspected deep vein thrombosis. Thromb Res.

[5] Perrier A (2000). Deep vein thrombosis and pulmonary embolism: a single disease entity with different risk factors?. Chest.

[6] Moser KM, Fedullo PF, LitteJohn JK, Crawford R (1994). Frequent asymptomatic pulmonary embolism in patients with deep venous thrombosis. JAMA.

[7] May R, Thurner J (1957). The cause of the predominantly sinistral occurrence of thrombosis of the pelvic veins. Angiology.

[8] Narayan A, Eng J, Carmi L (2012). Iliac vein compression as risk factor for left- versus right-sided deep venous thrombosis: case-control study. Radiology.

[9] Goodacre S, Sutton AJ, Sampson FC (2005). Meta-analysis: The value of clinical assessment in the diagnosis of deep venous thrombosis. Ann Intern Med.

[10] Wells PS, Owen C, Doucette S, Fergusson D, Tran H (2006). Does this patient have deep vein thrombosis?. JAMA.

[11] Constans J, Nelzy ML, Salmi LR (2001). Clinical prediction of lower limb deep vein thrombosis in symptomatic hospitalized patients. Thromb Haemost.

[12] Wells PS, Anderson DR, Rodger M (2003). Evaluation of D-dimer in the diagnosis of suspected deep-vein thrombosis. N Engl J Med.

[13] Wells PS, Anderson DR, Bormanis J (1997). Value of assessment of pretest probability of deep-vein thrombosis in clinical management. Lancet.

[14] Constans J, Boutinet C, Salmi LR (2003). Comparison of four clinical prediction scores for the diagnosis of lower limb deep venous thrombosis in outpatients. Am J Med.

[15] Subramaniam RM, Snyder B, Heath R, Tawse F, Sleigh J (2006). Diagnosis of lower limb deep venous thrombosis in emergency department patients: performance of Hamilton and modified Wells scores. Ann Emerg Med.

[16] Perone N, Bounameaux H, Perrier A (2001). Comparison of four strategies for diagnosing deep vein thrombosis: a cost-effectiveness analysis. Am J Med.

[17] Anderson DR, Kovacs MJ, Kovacs G (2003). Combined use of clinical assessment and d-dimer to improve the management of patients presenting to the emergency department with suspected deep vein thrombosis (the EDITED Study). J Thromb Haemost.

[18] Schutgens RE, Ackermark P, Haas FJ (2003). Combination of a normal D-dimer concentration and a non-high pretest clinical probability score is a safe strategy to exclude deep venous thrombosis. Circulation.

[19] Cademartiri F, Nieman K, van der Lugt A (2004). Intravenous contrast material administration at 16-detector row helical CT coronary angiography: test bolus versus bolus-tracking technique. Radiology.

[20] Hartmann IJ, Prokop M (2005). Pulmonary embolism: is multislice CT the method of choice?. For Eur J Nucl Med Mol Imaging.

[21] Schuemichen C (2005). Pulmonary embolism: is multislice CT the method of choice?. Against Eur J Nucl Med Mol Imaging.

[22] Johnson TR, Krauss B, Sedlmair M (2007). Material differentiation by dual energy CT: initial experience. Eur Radiol.

[23] Habis M, Paul JF (2010). Multidetector computed tomography of right ventricular acute myocardial infarction. Arch Cardiovasc Dis.

[24] Frederick MG, Hertzberg BS, Kliewer MA (1996). Can the US examination for lower extremity deep venous thrombosis be abbreviated? A prospective study of 755 examinations. Radiology.

[25] Theodoro D, Blaivas M, Duggal S, Snyder G, Lucas M (2004). Real-time B-mode ultrasound in the ED saves time in the diagnosis of deep vein thrombosis (DVT). Am J Emerg Med.

[26] Zhou C, Chan HP, Sahiner B (2007). Automatic multiscale enhancement and segmentation of pulmonary vessels in CT pulmonary angiography images for CAD applications. Med Phys.

